# Diamagnetic Composites for High‐Q Levitating Resonators

**DOI:** 10.1002/advs.202203619

**Published:** 2022-09-30

**Authors:** Xianfeng Chen, Satya K. Ammu, Kunal Masania, Peter G. Steeneken, Farbod Alijani

**Affiliations:** ^1^ Department of Precision and Microsystems Engineering Delft University of Technology Mekelweg 2 Delft 2628 CD The Netherlands; ^2^ Shaping Matter Lab Faculty of Aerospace Engineering Delft University of Technology Delft 2629 HS The Netherlands; ^3^ Kavli Institute of Nanoscience Delft University of Technology Lorentzweg 1 Delft 2628 CJ The Netherlands

**Keywords:** composites, diamagnetic levitation, eddy current damping, quality factor

## Abstract

Levitation offers extreme isolation of mechanical systems from their environment, while enabling unconstrained high‐precision translation and rotation of objects. Diamagnetic levitation is one of the most attractive levitation schemes because it allows stable levitation at room temperature without the need for a continuous power supply. However, dissipation by eddy currents in conventional diamagnetic materials significantly limits the application potential of diamagnetically levitating systems. Here, a route toward high‐*Q* macroscopic levitating resonators by substantially reducing eddy current damping using graphite particle based diamagnetic composites is presented. Resonators that feature quality factors *Q* above 450 000 and vibration lifetimes beyond one hour are demonstrated, while levitating above permanent magnets in high vacuum at room temperature. The composite resonators have a *Q* that is >400 times higher than that of diamagnetic graphite plates. By tuning the composite particle size and density, the dissipation reduction mechanism is investigated, and the *Q* of the levitating resonators is enhanced. Since their estimated acceleration noise is as low as some of the best superconducting levitating accelerometers at cryogenic temperatures, the high *Q* and large mass of the presented composite resonators positions them as one of the most promising technologies for next generation ultra‐sensitive room temperature accelerometers.

## Graphical abstract

Graphite particle based diamagnetic composites provide new routes for levitating macroscopic objects with ultralow damping in high vacuum. These levitating composites feature *Q*‐factors above 450 000 and life time beyond 1 h, while levitating over permanent magnets. Their high‐*Q* and large mass position them as one of the most promising technologies for next generation ultra‐sensitive room temperature accelerometers.

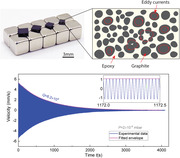

## 1 Introduction

The low dissipation and high quality factor (*Q*) of mechanical resonators makes them the devices of choice in precision time‐keeping, frequency filtering, and sensing applications. With the emergence of nano‐ and micro‐electromechanical systems, and the drive toward quantum limited mechanical elements, pushing the performance boundaries of resonators has become a matter of high scientific and societal relevance.^[^ ^1^, ^2^, ^3^, ^4^, ^5^, ^6^ ^]^ In particular, mechanical energy loss via the clamping points has become a dominant factor, limiting the *Q* of these resonators. As a consequence, attention has moved toward the field of levitodynamics.^[^ ^7^, ^8^ ^]^ By employing levitating resonators that are well isolated from their environment, losses can be minimized and extreme sensitivities can be achieved.

Optically, superconducting, and electrically levitating micro and nanoresonators have been shown to feature high *Q*s in the range 10^6^ − 10^7^.^[^ ^9^, ^10^, ^11^, ^12^ ^]^ Although these techniques are of great interest for fundamental studies, the requirement for continuous position control and cooling power supply^[^ ^8^ ^]^ narrows their application range, since the levitating object will collapse in a situation of power loss. Diamagnetic levitation is the only known method for realizing stable continuous vacuum levitation of objects at room temperature without external power supply.^[^ ^13^, ^14^, ^15^, ^16^ ^]^ Moreover, unlike optical and electrical levitation that are limited to nano‐gram objects,^[^ ^17^, ^18^ ^]^ diamagnetic levitation is the method of choice for levitating macroscopic objects whose larger mass can significantly enhance the sensitivity of sensors, like accelerometers^[^ ^19^ ^]^ and gravimeters.^[^ ^20^, ^21^, ^22^, ^23^ ^]^ However, the *Q* of conventional diamagnetic materials such as graphite that has high magnetic susceptibilities is significantly limited by eddy current damping forces.^[^ ^15^ ^]^ While the diamagnetic levitation of non‐conductive materials such as silica could make the levitodynamic system immune to the presence of eddy current damping forces, their magnetic susceptibility is lower, such that it normally only is suitable for levitating microscopic objects.^[^ ^24^, ^25^ ^]^

Here, we demonstrate millimeter scale composite plates comprising graphite microparticles dispersed in epoxy resin that levitate stably above permanent magnets and exhibit *Q*s above 450 000. The strong diamagnetic susceptibility of the graphite particles allows passive levitation of the composite plates, while the epoxy acts as an insulating material that suppresses eddy currents. To investigate the dependence of *Q* on composite properties, we perform simulations and experiments on composites with different particle sizes and volume fractions. We confirm that by reducing particle size, damping can be significantly decreased while maintaining the macroscopic size of the levitating object. Finally, we compare the performance of the realized diamagnetic composite resonator to state‐of‐the‐art accelerometers and show that it leads to one of the lowest acceleration noise figures achieved thus far in levitating sensors.

## 2 Results

### 2.1 Diamagnetically Levitating Composites

**Figure 1:**
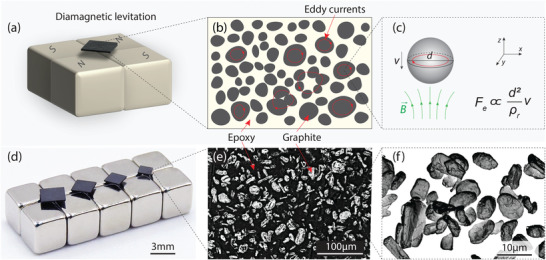
a) Schematic of a diamagnetic plate levitating above 4 cubic Nd_2_Fe_14_B magnets with alternating magnetization. b) Schematic of the eddy currents (red circular arrows) generated inside the graphite microparticles that are distributed in the composite. c) The relationship between the eddy current damping force *F*_e_ and particle size *d*, for a spherical particle with electrical resistivity *ρ*_r_ moving in a magnetic field (see Section S5,Supporting Information, for details). d) An array of graphite‐epoxy composite plates of different sizes levitating above magnets at room temperature and pressure. e) Confocal microscopy image of the surface of the composite plate with particle size of 17.6 um and volume fraction of 0.21, showing the distribution of the graphite particles (white) in the epoxy (black). f) Scanning electron microscopy image showing the size and morphology of the graphite particles.

To realize diamagnetically levitating resonators with high *Q*s, we fabricate composite materials with distributed graphite microparticles by dispersing them in epoxy resin through mechanical mixing, and then curing the resin in an oven (see Exprimental Section and Section S1, Supporting Information). The fabrication process enables a high degree of freedom in size of graphite particles and selection of resin composition. Due to the strong diamagnetic susceptibility of graphite, the composite levitates stably above permanent Nd_2_Fe_14_B magnets arranged in a checkerboard configuration with alternating magnetization (see **Figure** **1**a). We expect that the epoxy between the microparticles acts as an insulator, confining eddy currents within the particles (Figure 1b), and thus diminishing eddy current damping forces and increasing *Q*.^[^ ^15^ ^]^ Furthermore, since for a composite with particle size *d* moving in a magnetic field, the eddy current damping force per volume scales quadratically with particle size (*F*_e_∝*d* ^2^ see Figure 1c and Section S5, Supporting Information^[^ ^26^ ^]^), we expect that by reducing the microparticle size in the composite, high mechanical *Q*s can be achieved while maintaining macroscopic mass. To experimentally investigate this effect, square graphite/epoxy composite plates of different size with a constant 90 µm thickness are prepared, as shown in Figure 1d. The successful levitation of the composite plates with graphite volume fraction *V*_f_ of 21%, as shown in Figure 1d, confirms that the diamagnetism of graphite is maintained in the microparticles and that the diamagnetic force remains strong enough to oppose the gravitational force, even though the graphite particles have anisotropic magnetic susceptibilities and are randomly oriented inside the epoxy matrix. In Figure 1e,f, we show microscopic images of the composite and graphite microparticles from which we note that the particle sizes are distributed over a wide range (see the particle size measurement in Section S2.1, Supporting Information). Moreover, we quantitatively analyze the particle distribution (see Section S2.2, Supporting Information) and observe that the graphite particles are randomly distributed inside the epoxy matrix.

### 2.2 Q‐Factor Measurement

**Figure 2:**
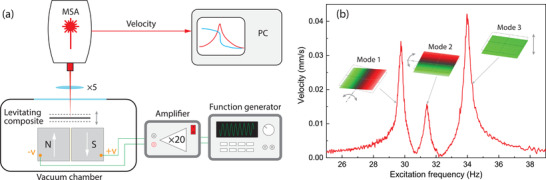
Experimental setup and rigid body dynamic response of a levitating composite resonator. a) Schematic of the measurement setup comprising a MSA400 Polytec Laser Doppler Vibrometer (LDV) for the readout and electrostatic force as the actuation means. The drive voltage is generated by the function generator and is amplified by a 20× voltage amplifier that drives the levitating plate into resonance. The electrostatic force is generated by applying voltage between the magnets beneath the levitating plate. By focusing the vibrometer's laser beam on the plate, the plate motion is captured, and the acquired velocity is used for spectral analysis. b) The frequency response curve of a 1.8 × 1.8 × 0.09 mm^3^ levitating composite plate with 8.6 µm graphite particles measured at 0.1 mbar. Three of the measured mode shapes using LDV are shown close to the corresponding resonance peaks.

To probe the vibrations of the levitating plates, we use a Polytec MSA400 Laser Doppler Vibrometer (LDV) and measure their out‐of‐plane velocity in a vacuum chamber at a pressure of 0.1 mbar (see **Figure** **2**a and Experimental Section). We characterize the spectral response of the levitating objects by driving them electrostatically at different frequencies. Figure 2b shows the area‐averaged magnitude of the spectral response for a 1.8 × 1.8 × 0.09 mm^3^ composite plate with 8.6 µm graphite particles. Three plate resonance peaks can be identified in the spectral response, which correspond to the two rotational modes at 29.7 Hz (Mode 1) and 31.4 Hz (Mode 2) and the translational rigid body mode of vibration at 34.0 Hz (Mode 3). In this work, we focus on the *Q* of the out‐of‐plane translational mode that relates to the vertical motion (Mode 3). The mode shapes are identified by scanning the laser over the plate surface at the corresponding resonance frequencies, and are shown in Figure 2b.

**Figure 3:**
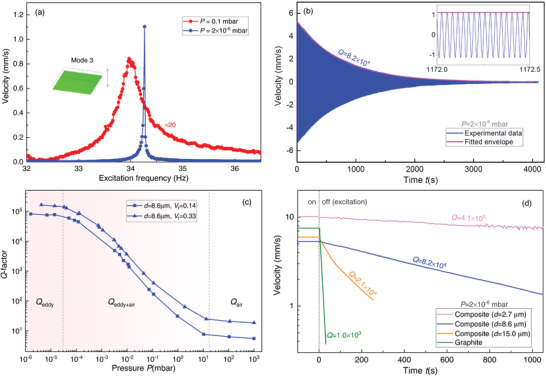
Energy dissipation measurements. a) Frequency response curves for the translational mode of the 1.8 × 1.8 × 0.09 mm^3^ levitating composite plate measured at 0.1 mbar and 2 × 10^−6^ mbar. The frequency response curve at 0.1 mbar has been multiplied by a factor of 20 for visibility. b) Undriven ringdown of the same composite plate for a duration of 4,000 s at 2 × 10^−6^ mbar and its fitted envelope. The time signal for a 0.5 s interval is also shown in the inset. c) The *Q* as a function of pressure for two sizes of composite plate shows three characteristic regions comprising the region where air damping is dominant (right), region where both air and eddy current damping contribute to dissipation (middle), and region where eddy current damping is dominant (left). d) Ringdowns of a levitating graphite plate and three composite plates composed of different particle size, revealing that decreasing the particle size results in higher *Q* of the samples. The dashed line separates the time span between the excitation is on and off.

Since eddy current and air damping^[^ ^15^ ^]^ are the major sources of dissipation in diamagnetically levitating objects, we minimize the effect of air damping by operating the composite plate resonator in high vacuum (10^−6^ mbar). In **Figure** **3**a, we compare the resonant response of the plate's translational mode in low (0.1 mbar) and high (10^−6^ mbar) vacuum environments. We find an increase in the resonance frequency that we attribute to a reduction in mass loading by the surrounding gas. Moreover, the high vacuum results in a much sharper peak, with much higher *Q*, due to the reduction of air damping effects. In fact, the *Q* is so high that it is difficult to accurately determine it using a frequency response measurement, due to the limited resolution bandwidth of the measurement setup.

To determine the *Q* more accurately while also minimizing the influence of spectral broadening, we perform ringdown measurements. These are conducted by first electrostatically exciting the composite plate at its resonance frequency, then switching off the excitation voltage and recording the free vibration decay. The amplitude of the underdamped vibration decays proportional to $\propto e^{-\frac{t}{\tau }}$, where $\tau =\frac{Q}{\pi f_{\mathrm{res}}}$ is the decay constant, and *f*_res_ is the resonance frequency of the plate. In Figure 3b, we show a typical measurement for the translational mode of the levitating composite. Note that a very long vibration lifetime of ≈4, 000 s is observed, corresponding to a *Q* of 8.2 × 10^4^. In the inset of Figure 3b, we also show the free vibrations of the plate over a 0.5 s time interval, demonstrating a clear sinusoidal response during the energy decay measurements. It is noted that due to the presence of low‐frequency perturbations from the vacuum pump and the environment, the amplitude of the high‐*Q* composites might fluctuate during the ringdown measurements as shown in Figure 3d. However, the fluctuations do not influence the *Q* factor measurements as they are very small compared to the vibration amplitude.

To ensure that the energy decay constant τ is not limited by air damping, we sweep the pressure from 10^−6^ − 10^3^ mbar and measured *Q* as a function of air pressure (see Figure 3c). The data for two composite plates with *d* = 8.6 µm particle size and different graphite volume fractions show three distinct regions in the *Q* versus pressure plot. When the pressure is reduced below 3 × 10^−5^ mbar, *Q* reaches a plateau, as shown in Figure 3c. This suggests that air damping has become negligible, and *Q* is solely limited by eddy currents. The *Q*s shown in the rest of this work are measured at a pressure below 5×10^−6^ mbar to eliminate the effect of air damping in our measurements.

### 2.3 Tailoring Composite Properties to Suppress Eddy Currents

To investigate the effect of the graphite particle size and volume fraction on the levitation forces and the eddy current damping, we fabricate square plates with different graphite volume fractions *V*_f_, side length *L*, and particle size *d*. In Figure 3d, we compare the ringdown response of three 1.8 × 1.8 × 0.09 mm^3^ graphite composite plates with different particle sizes, namely *d* = 15.0, 8.6 and 2.7 µm. We find that the plate that encompasses the smallest particle size exhibits the largest value of *Q*. Remarkably, we observe an increase of nearly 410 times in *Q* for the 2.7 µ m particle composite plates compared to the levitating graphite plate.

To better understand this observation and gain deeper insight into the mechanisms accountable for *Q* enhancement, simulations based on Finite Element Method (FEM) are performed to calculate the levitation height and eddy current damping force using COMSOL multiphysics. These simulations are carried out assuming that the graphite particles have a spherical shape and are distributed inside the matrix (see Section S3, Supporting Information, for details of the numerical modeling and parameter values used in our simulations). We note that graphite is inherently anisotropic.^[^ ^13^ ^]^ However, in our fabrication procedure, graphite particles are randomly oriented in the epoxy matrix, and thus by considering all possible orientations in the matrix, the local anisotropy can be averaged out and the effective macroscopic behavior can be viewed isotropic. For this reason, in our study, we treat the magnetic susceptibility of graphite as an effective value χ_eff_ that we evaluate by fitting our FEM simulations to the measured levitation height of the composite from experiments (see Figure S8, Supporting Information, for more details).

**Figure 4:**
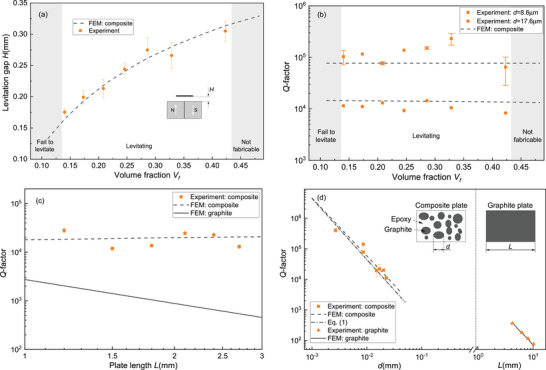
Dependence of the levitation force and dissipation on composite particle and plate size. a) Levitation gap *H* of the 1.8 × 1.8 × 0.09 mm^3^ plate with 17.6 µm particles as a function of volume fraction. b) *Q*‐factor of two 1.8 × 1.8 × 0.09 mm^3^ plates with different particle size as a function of volume fraction. The gray area in Figure 4a,b represents the volume fraction of which the composites can not be levitated. c) *Q*‐factor of square composite plates with a thickness of 90 µm as a function of plate side length *L*. The composite plates are made from *d* = 17.6 µm particles with 0.21 volume fraction. d) Dependence of *Q* on particle size. The left side of the graph (before the short dashed line) shows the *Q* of composite plates with varying graphite particle size. Since the *Q* is only weakly dependent on the volume fraction and side length (see Figure 4b,c), the error bars in the data on the left side of Figure 4d are obtained by analyzing the *Q*s obtained from plates with a thickness of 90 µm but different side lengths (1.2–2.7 mm) and volume fraction (0.14–0.32) at fixed *d*. The right side of the graph (after the short dashed line) shows the experimental *Q* of levitating plates made of pyrolytic graphite with 0.28 mm thickness and different side lengths *L* on the *x*‐axis. The insets show schematics of the composite and graphite plate. In Figure 4, the dashed and solid lines correspond to the FEM simulations for composite and graphite plates as described in Section S3 (Supporting Information), respectively. Moreover, the dashed‐dotted line in Figure 4d represents the *Q*s obtained from Equation (1). The dots represent experimental data.

In **Figure** **4**a, we show the levitation height of the composite plates with particle size *d* = 17.6 µm as a function of volume fraction *V*_f_. We find that composites with a graphite volume fraction below 14% (*V*_f_ < 0.14) do not provide sufficient diamagnetic force to counteract gravity and thus do not levitate. For composites with a graphite volume fraction above 43% (*V*_f_ > 0.43), the samples cannot be produced with sufficient structural integrity due to the high particle content. Between these two limits, we observe a steady increase in the levitation height that agrees well with the simulations. These results indicate that the increase of the magnetic force is dominant over the increase in the overall gravitational force through the higher mass density of the graphite particles compared to the epoxy, see Table SII in Section S3 (Supporting Information).

We also study the influence of volume fraction *V*_f_ on the measured *Q* for composite plates with particle sizes *d* = 8.6 µm and *d* = 17.6 µm, as shown in Figure 4b. It is interesting to see that unlike levitation height, *Q* does not significantly change with *V*_f_, even though the measured bulk conductivity reveals an increase in the conductivity with the increase of *V*_f_ (see Section S4, Supporting Information). This result suggests that the variations in bulk conductivity do not contribute considerably to the observed dissipation. A similar effect is seen in Figure 4c, where we show the experimentally obtained *Q* of square plates with different side lengths *L*, that are cut out of the same composite with *d* = 17.6 µm and *V*_f_ = 0.21. It is observed from the figure that a reduction in side length does not substantially increase *Q*. This observation contrasts with *Q*s estimated from COMSOL simulations for pyrolytic graphite plates that increase close to an order of magnitude with reducing *L*.^[^ ^15^ ^]^

The volume fraction and plate size independent *Q*s obtained from both experiments and simulations in Figure 4b,c indicate that the majority of eddy current damping occurs inside the graphite particles and is not caused by currents flowing in between them. Thus, increasing the particle density increases the stored kinetic energy *E*_k_ (proportional to the mass) by approximately the same factor as the eddy current dissipation *E*_d_ (proportional to the number of particles) (see Figure S9 in Section S3.2, Supporting Information), such that the *Q*, which is proportional to their ratio *E*_k_/*E*_d_, remains nominally constant.

After having investigated the effect of volume fraction and composite plate size, we now investigate the effect of graphite particle size *d* on *Q*. It can be observed from both the experimental and numerical results (see left side of Figure 4d) that reducing the particle size *d* results in a clear increase in the *Q* of the composite plates. The *Q* increases from ≈ 10 000 at *d* = 22.7 µm to a value as high as 460 000 at *d* = 2.7 µm, which is to our knowledge a record value for passively levitating diamagnetic resonators at room temperature. On the right side of Figure 4d, the *Q*s of pyrolytic graphite plates with varying side lengths are shown. These plates also show an increasing *Q* with decreasing plate size.^[^ ^15^ ^]^

## 3 Discussion and Conclusions

To understand these findings, and in particular the increase in *Q* as a function of *d*, we use Faraday's law and obtain an analytic expression for the *Q* of a graphite/epoxy composite plate that moves in a magnetic field (see Section S5, Supporting Information, for the detailed derivation):

(1) $$Q=\frac{80\pi f_{\mathrm{res}}\rho_{r}\left(\left(\rho_{g}-\rho_{e}\right)+\rho_{e}/V_{f}\right)}{{\left(C_{r}d\right)}^{2}\nabla^{2}B},$$

where *ρ*_r_ is the resistivity, *ρ*_g_ is the density of graphite, *ρ*_e_ is the density of epoxy, and *C*_r_ is the effective particle size factor that we use to account for experimental deviations from the theoretical model due to variations in particle size, composition, morphology and distribution. Moreover, ∇^2^ *B* represents the Laplacian of the magnetic field, which is

(2) $$\nabla^{2}B=\frac{\int_{V_{\mathrm{plate}}}{\left(\frac{dB}{dz}\right)}^{2}dV_{\mathrm{plate}}}{V_{\mathrm{plate}}}.$$

To compare our experimental findings in Figure 4d to the analytical expression Equation (1), we take *f*_res_ = 35 Hz, *ρ*_r_ = 5 × 10^−6^ Ω*m*, *ρ*_g_ = 2260 kg m^−3^, *ρ*_e_ = 1100 kg m^−3^, and use COMSOL simulations to calculate ∇^2^ *B* = 1.1 × 10^6^ (T/m)^2^ for a 1.8 × 1.8 × 0.09 mm^3^ plate that levitates 0.26 mm above the magnets, corresponding to a composite with *V*_f_ = 0.32 (see Figure 4a). Using these values and *C*_r_ = 6.3 as a fit parameter, we can match the experimental data shown in Figure 4d with good accuracy. These results show that the *Q* in our levitating composites is inversely proportional to *d* ^2^, providing evidence that the strong dependence of *Q* on particle size can be mainly accounted for using Equation (1), which is based on dissipation due to eddy currents that flow inside the graphite particles. The high sensitivity of *Q* to *d* allows us to engineer and increase the *Q* of our levitating resonators by using different particle size while keeping the macroscopic dimensions of the plate constant. The highest *Q* we obtain with this fabrication process is 4.6 × 10^5^ for a 2.7 × 2.7 × 0.09 mm^3^ composite plate with *d* = 2.7 µm particles and *V*_f_ = 0.21 volume fraction, which is two orders of magnitude higher than a pyrolytic graphite plate of the same size as shown in Figure 4d.

It is of interest to note that extrapolation of the graphite plate data in the right side of Figure 4d to smaller values of *d* leads to much higher values of *Q* than that are obtained experimentally with the composites in the left part of Figure 4d. Several mechanisms might account for this difference, including the random orientation of the graphite particles in the composite, the particle size and shape variations, inactive layers on the particle surfaces and material parameter differences between the graphite in the plates and particles. In Figure 4d, the combined effect of these mechanisms are captured by the effective particle size factor *C*_r_. Although we cannot fully account quantitatively for the relatively large value of *C*_r_ = 6.3 of this factor, possibly a small fraction of larger particles or clusters of particles in the composite accounts for a large part of the damping force. Microscopic images of the composites in Section S2 (Supporting Information) support this hypothesis, by showing that the dispersion of the particles is random and less homogeneous inside the epoxy matrix with local particle clusters. It might also be that not all sources of damping are included in Equation (1) and more sophisticated models will need to be developed. Nevertheless, we foresee that by further control of the particle size and optimization of its distribution, levitating composites can achieve *Q*s above 1 million for millimeter composites with 1 µm or smaller particles.

**Figure 5:**
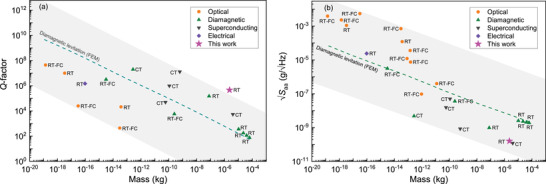
a) *Q*‐factor versus mass of different levitating systems (optical,^[^ ^9^, ^10^, ^27^, ^28^, ^29^ ^]^ diamagnetic,^[^ ^15^, ^24^, ^25^, ^30^, ^31^ ^]^ superconducting^[^ ^11^, ^19^, ^32^, ^33^ ^]^ and electrical^[^ ^12^ ^]^). b) Plot of acceleration noise floor against mass of different levitating accelerometers (optical,^[^ ^9^, ^10^, ^17^, ^27^, ^28^, ^29^, ^34^, ^35^, ^36^, ^37^, ^38^ ^]^ diamagnetic,^[^ ^15^, ^24^, ^25^, ^30^, ^31^ ^]^ superconducting^[^ ^11^, ^19^, ^32^, ^33^ ^]^ and electrical^[^ ^12^ ^]^). RT stands for *Q*s measured at room temperature without feedback cooling, CT stands for *Q*s measured at cryogenic temperature without feedback cooling, RT‐FC stands for natural *Q*s estimated from feedback cooling measurements. The *Q* and $\sqrt{S_{\mathrm{aa}}}$ of different levitating systems are also shown in Table SIII–SV of Section S6 (Supporting Information). The *Q* and $\sqrt{S_{\mathrm{aa}}}$ shown as dashed lines are simulated using COMSOL for graphite plates with different size *L* (detailed material parameter values used for these simulations can be found in Table SI of Section S3, Supporting Information). In the simulations, the plate thickness *t* and magnet size *D* are taken proportional to the plate side length (*D* = 1.2*L* and *t* = 0.03*L*). The grey area in Figure 5a,b sets the boundary of the *Q* and acceleration noise floor against mass of available levitodynamic systems in the literature.

The combination of high *Q* and large mass of the levitating composites promises low noise floor levels in accelerometry. In **Figure** **5**, we benchmark the presented levitating composite plates against state‐of‐the‐art levitodynamic systems by plotting mass against *Q* (Figure 5a) and the square root of the acceleration noise power spectral density *S*_*aa*_∝*f*_res_/(*mQ*)^[^ ^19^ ^]^ (Figure 5b), which is a measure of the limit of detection of an accelerometer. The plots compare a range of superconducting, diamagnetically, electrically, and optically levitating systems at room temperature (labeled with RT in Figure 5), at cryogenic temperature (CT) or using feedback cooling (RT‐FC). Note that RT‐FC stands for natural *Q*s that are estimated from feedback cooling measurements. The plots also show the theoretical estimates of *Q* and $\sqrt{S_{aa}}$ (dashed lines) as a function of mass for diamagnetically levitating pyrolytic graphite. It appears from this benchmark that in terms of acceleration noise floor and *Q*, diamagnetic composites stand out, providing the possibility to levitate large, high‐*Q* objects using relatively weak fields from permanent magnets. The combination of large levitating proof mass and high *Q* make these composites attractive materials for realizing next generation room temperature accelerometers with theoretical sensitivities as low as $0.16ng/\sqrt{Hz}$, that are comparable to superconducting levitodynamic systems at cryogenic temperatures (Figure 5b).

In conclusion, we demonstrate diamagnetic high‐*Q* composite plate resonators consisting of graphite particles dispersed in an epoxy matrix that can be levitated at room temperature above permanent magnets with graphite volume fractions as low as 14%. By insulating the graphite particles, eddy currents are reduced and confined within the particles, allowing us to suppress the associated damping forces. This enables a remarkable enhancement in *Q*, reaching values as high as nearly 0.5 million at room temperature. Measurements of the dependence of damping to particle volume fraction, plate length, and particle size are compared to FEM models, and show good agreement with an analytical model for eddy current damping forces that predicts *Q* to be inversely proportional to the squared particle size *Q*∝1/*d* ^2^. Reduction of the particle size and optimization of particle distribution and orientation, can lead to novel composites that further enhance the performance of future macroscopic levitating devices used as accelerometers,^[^ ^19^ ^]^ gravimeters,^[^ ^20^, ^21^, ^22^, ^23^ ^]^ or sensors for exploring macroscopic limits of quantum mechanics.^[^ ^5^, ^39^, ^40^, ^41^ ^]^

## 4 Experimental Section

### Composite Fabrication

Graphite micro powders (purity > 99.9%) with mean sizes from 2.7 to 22.7 µm were purchased from Nanografi Nano Technology. Particle size distribution measurements were performed using Malvern Mastersizer 3000 on 0.1% weight/volume aqueous solution of the powders using sodium dodecyl sulfate solution as surfactant. The particle size *d* of each type of powder was represented by the mean value of the distribution. The morphology of these powders was confirmed via Scanning Electron Microscopy (JEOL JSM‐7500F).

The details of the graphite composite fabrication process are shown in Figure S1 (Supporting Information). First, the two components of the epoxy (Epotek 302‐3M from Gentec Benelux) were mixed at 3500 rpm for 5 min in a Dual Asymmetric Centrifuge mixer (DAC 150.1 FVZ‐K ) followed by the addition and mixing of the graphite powder at 500 rpm for 5 min. To reduce the viscosity of the resulting graphite‐epoxy paste, ethanol was added, and further mixed at 500 rpm for 5min. This maximized dispersion and homogeneity of the paste with the graphite particles in the epoxy‐ethanol matrix. The paste was then transferred into circular holes (*ϕ* = 10mm) in a thin plastic mould with thickness of 0.12 mm on the top of a flat steel mould. The deposited paste was left at room temperature and pressure for 30 min to let the ethanol fully evaporate before curing the epoxy in order to minimize porosity. The graphite/epoxy paste was then compressed by steel moulds and cured in an oven at 100°C for ≈ 12 h. After curing, an Optec micro laser cutter was used to cut the composite into square plates with desired lengths. Finally, fine sand paper (5 µm grain) was used to polish composite surface to the desired thickness.

### Measurement

In our experiments, the excitation voltage was generated by the Polytec MSA400 vibrometer for the resonance frequency measurements, and by a function generator for the ringdown response measurements. The electrostatic force was generated as shown in Figure 2a, by applying a voltage difference between the magnets beneath the levitating plate. To isolate the magnets from one another, Kapton tape was used. When a voltage was applied between the two electrodes, the levitating plate acted as a floating electrode between the two electrodes, thereby forming a capacitive divider. In the area at which the plate overlaped with the electrodes, an electrostatic downward force was exerted that depended on the overlap area, voltage difference, and gap size. Since the electrostatic force was proportional to the square of the voltage, a DC offset voltage was added to make sure the electrostatic force had a component of the same frequency as the output voltage. Finally, to read out the motion, a Polytec LDV was used. The LDV measurements were conducted in a vacuum chamber over a pressure range of 10^−6^ − 1000 mbar at room temperature.

## Conflict of Interest

The authors declare no conflict of interest.

## Supporting information

Supporting Information

## Data Availability

The data that support the findings of this study are available from the corresponding author upon reasonable request.
