# Supplementary material for: Diamagnetic Composites for High‐Q Levitating Resonators
Source: Adv Sci (Weinh). 2022 Sep 30;9(32):2203619. doi: 10.1002/advs.202203619 (PMC9661851; doi:10.1002/advs.202203619)
Supplement: Supplementary file 1 — Supporting Information [file ADVS-9-2203619-s001.pdf]

Supporting Information: Diamagnetic composites for high-Q levitating resonators

Xianfeng Chen,[1] Satya Ammu,[2] Kunal Masania,[2] Peter G. Steeneken,[1,3] and Farbod Alijani[1]

[1] *Department of Precision and Microsystems Engineering, Delft University of Technology, Mekelweg 2, 2628 CD, Delft, The Netherlands*

[2] *Shaping Matter Lab, Faculty of Aerospace Engineering, Delft University of Technology, 2629 HS, Delft, The Netherlands*

[3] *Kavli Institute of Nanoscience, Delft University of Technology, Lorentzweg 1, 2628 CJ, Delft, The Netherlands.*

(Dated: September 19, 2022)

In S1, we show the details of graphite composite fabrication process. S2 details out particle size measurement (S2.1) and particle dispersion analysis (S2.2). S3 presents the FEM methodology used to calculate the eddy current damping in a graphite (S3.1) and a composite plate (S3.2). S4 shows the electrical conductivity measurements of composites with different volume fractions. In S5, we present the analytical model to calculate the eddy current damping of a composite plate moving in a magnetic field. Finally, in S6 we show the Qs and acceleration noise floor of state-of-the-art levitodynamic systems.

S1: COMPOSITE FABRICATION PROCESS

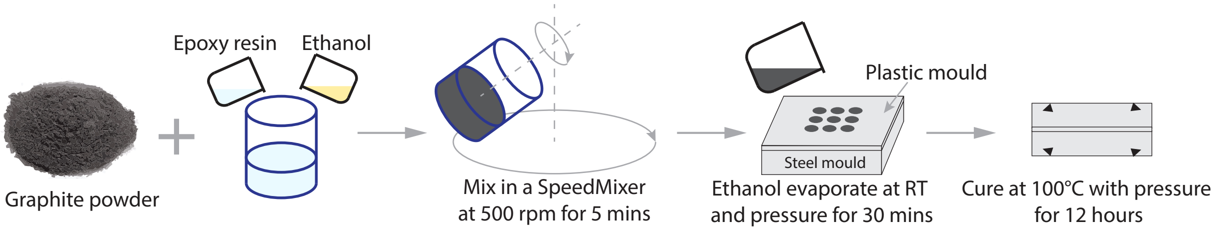

FIG. S1. The schematic of composite fabrication process.

The details of graphite composite fabrication process is shown in Fig. S1. First, the two components of the epoxy are mixed at 3500 rpm for 5 minutes in a Dual Asymmetric Centrifuge mixer (DAC 150.1 FVZ-K) followed by the addition and mixing of the graphite powder at 500 rpm for 5 minutes. To reduce the viscosity of the resulting graphite-epoxy paste, ethanol is added, and further mixed at 500 rpm for 5 mins. This maximises dispersion and homogeneity of the paste with the graphite particles in the epoxy-ethanol matrix. The paste is then transferred into circular holes ($\phi = 10\,\mathrm{mm}$) in a thin plastic mould with thickness of 0.12 mm on the top of a flat steel mould. The deposited paste is left at room temperature and pressure for 30 minutes to let the ethanol fully evaporate before curing the epoxy to minimise porosity. Afterwards, the graphite/epoxy paste is compressed by steel moulds with pressure, which is then cured in an oven at 100 °C for around 12 hours. Once the graphite/epoxy composite is cured, we use a micro laser cutter to cut the composite into square plates with desired lengths and use fine sand paper (5 µm grain) to polish its surface to the desired thickness.

S2: PARTICLE SIZE MEASUREMENT AND PARTICLE DISPERSION ANALYSIS

S2.1: Particle size measurement

Fig. S2 shows the size distribution of the graphite powders used in our experiments. The particle size distribution measurements are performed using Malvern Mastersizer 3000 on 0.1% w/v aqueous solution of the powders using sodium dodecyl sulfate solution as surfactant. The measurement of each type of powders is repeated five times. From this figure, it can be seen that the particle size of each type of powder has a wide range of distribution. In the main text, we use the mean value of the distribution to represent the particle size d.

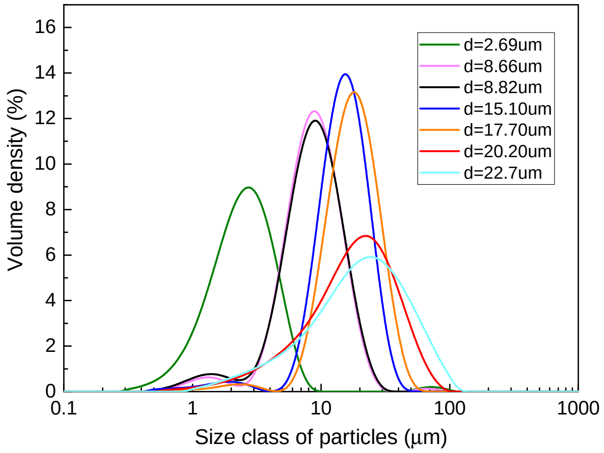

FIG. S2. Particle size distribution for different particles used in the experiments.

S2.2:Particle dispersion analysis

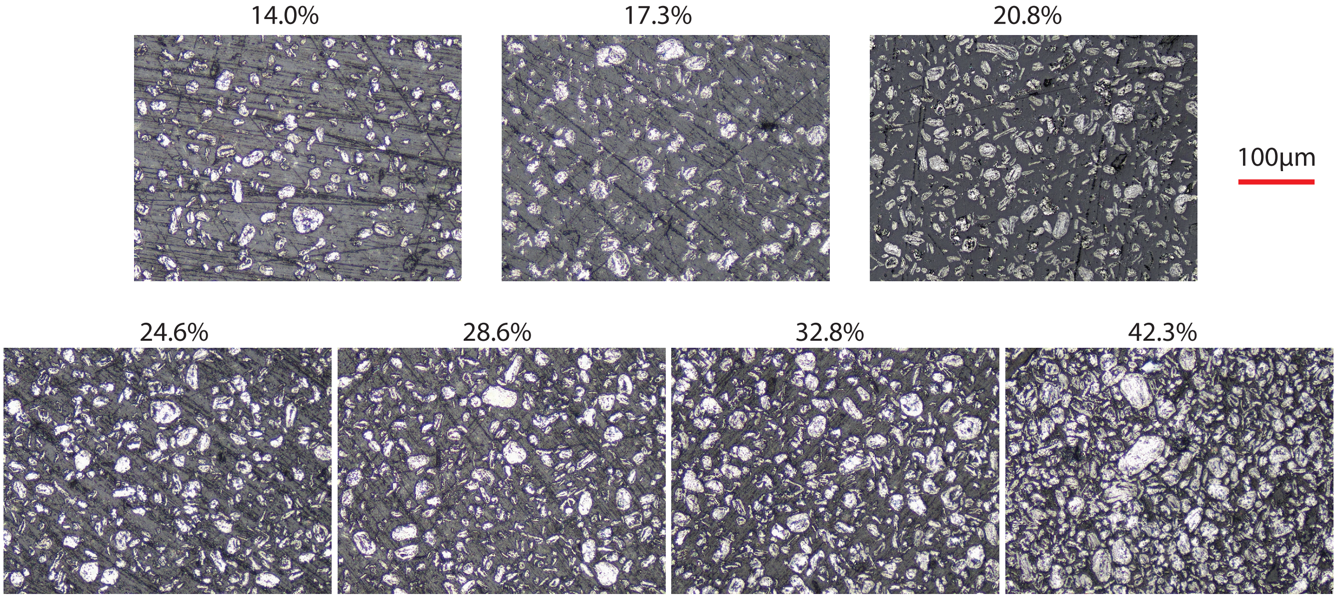

FIG. S3. Microscopic images of composites with 17.6 µm particles and different volume fractions.

Fig. S3 shows microscopic images of composites with 17.6 µm particles and different volume fractions. To obtain these images, samples are first polished with a fine sand paper to obtain a clear interface between the graphite particles and epoxy. The graphite particles are appearing in white color due to the reflection of light from the microscope. To quantify the dispersion quality of the particles inside epoxy, we use the Area Disorder (AD) of the Delaunay network as described in reference [1]. AD is a dimensionless quantity with values between 0 and 1. $AD = 0$ means the dispersion is perfect and particles are homogeneously distributed inside the matrix. $AD = 1$ means the dispersion is worst with clusters, as shown in Fig. S5. To obtain the AD, we first identify the particle boundaries (Fig. S4b) from the optical image (Fig. S4a), and then locate the particles' center of mass (Fig. S4c). Next, we build the Delaunay network (Fig. S4d) to calculate AD. Fig. S5 shows the AD for the composites in Fig. S3. From Fig. S5, we can see that the dispersion of our composites is random-like.

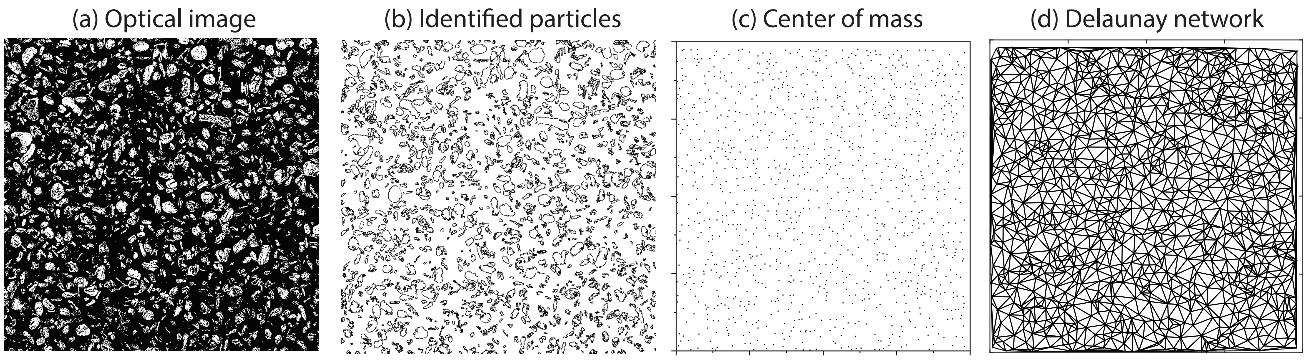

FIG. S4. Particle dispersion analysis on a composite with 17.6 µm particles and 20.8 % volume fraction. To obtain the Area Disorder AD, we first take an image of the composite (a) and then identify the particles (b) using ImageJ. Afterwards, the particles' center of mass (c) is located, from which we build their Delaunay network (d) to calculate the AD.

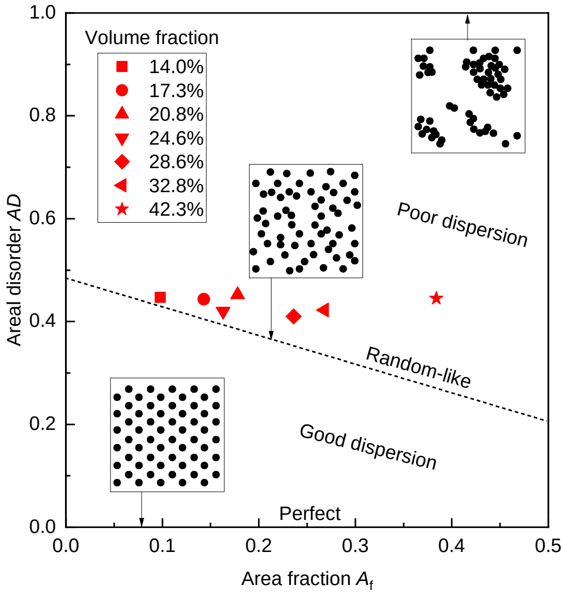

FIG. S5. Quantitative analysis of the particle dispersion in composites with different volume fractions. The Area fraction A_f is obtained via image processing.

S3: COMSOL SIMULATIONS FOR OBTAINING EDDY CURRENT DAMPING FORCES

S3.1. FEM: Eddy current damping in a graphite plate

This section details out the methodology we use to calculate the eddy current damping forces of a square graphite plate levitating above four permanent magnets. The geometry of the model is shown in Fig. S6.

We simulate the magnetic field using *COMSOL Multiphysics 5.6*. Assuming that the influence of the diamagnetic plate on the field is negligible, the integrated magnetic force on the plate, $\mathbf{F_B}$, can be determined using

$$\mathbf{F_B} = \boldsymbol{\nabla} \int_{\mathcal{V}} \mathbf{M} \cdot \mathbf{B} \mathrm{d}\mathcal{V} = \frac{\mu_0}{2} \int_{\mathcal{V}} \boldsymbol{\nabla} (\chi_x H_x^2 + \chi_y H_y^2 + \chi_z H_z^2) \mathrm{d}\mathcal{V}, \tag{1}$$

where χ_x, χ_y, χ_z are the magnetic susceptibility of the levitating plate in x, y, z directions, $\mathcal{V}$ is the volume of the plate, $\mathbf{B}$ is the magnetic field and $\mathbf{M}$ is the plate's magnetization. The components of the magnetic field inside the plate are $H_{x,y,z} = B_{x,y,z}/\mu$, where $\mu \approx \mu_0$ is the magnetic permeability of graphite. By calculating the magnetic force in z direction with different levitation gaps between the plate and magnets, the levitation height where the z-component of magnetic force is equal and opposite to the gravitational force is obtained. Using the levitation height, the stiffness

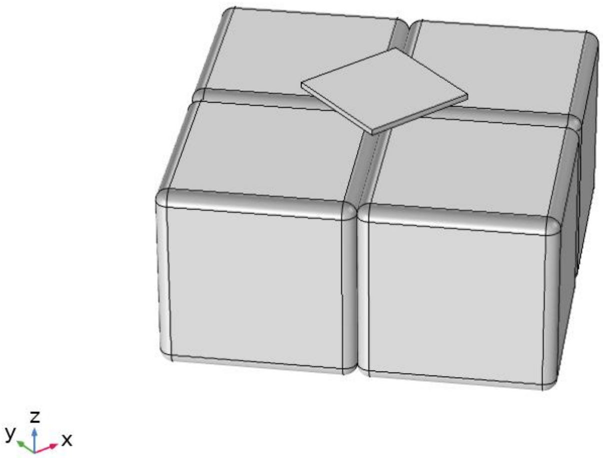

FIG. S6. Geometry model of magnets and graphite plate.

TABLE I. Material properties used for the simulations of the levitating pyrolytic graphite.

Property	Symbol	Value	Unit
Density	ρ	2070	kg/m^3
Susceptibility $\perp$ [2]	χ_z	-450	$\times 10^{-6}$
Susceptibility $\parallel$ [2]	$\chi_{x,y}$	-85	$\times 10^{-6}$
Conductivity $\perp$[3]	σ_z	200	S/m
Conductivity $\parallel$[3]	$\sigma_{x,y}$	200000	S/m

of the magnetic force k can then obtained by taking the derivative of F_z over z at the equilibrium point. Next, the resonance frequency $f_{\rm res}$ is calculated using $f_{\rm res} = \frac{1}{2\pi}\sqrt{\frac{k}{m}}$, where m is the mass of the plate.

Then, we simulate the eddy current damping of the plate. We note that when a conductor moves with velocity vector $\mathbf{v}$ through a magnetic flux density field $\mathbf{B}$, the charge carriers inside the conductor feel an electric field $\mathbf{v} \times \mathbf{B}$ due to the Lorentz' force in addition to the field from the electric potential V_e, that generates an eddy current density $\mathbf{J}$ given by

$$\mathbf{J} = -\sigma \nabla V_e + \sigma(\mathbf{v} \times \mathbf{B}), \tag{2}$$

where σ is the electrical conductivity. By combining Eq. (2) with the current continuity condition $\nabla \cdot \mathbf{J} = 0$ and the boundary condition $\mathbf{J} \cdot \mathbf{n} = 0$ ($\mathbf{n}$ is the unit vector perpendicular to the boundary), we determine the eddy current density distribution $\mathbf{J}$ numerically for known $\mathbf{v}$, σ, $\mathbf{B}$. We then evaluate the the total damping contribution due to eddy currents as follows

$$\mathbf{F_e} = \int_{\mathcal{V}} \mathbf{J} \times \mathbf{B} \mathrm{d}\mathcal{V}, \tag{3}$$

where integration is done over the graphite plate volume $\mathcal{V}$. Noting that the eddy current damping force $\mathbf{F}_{\rm e}$ is proportional and in the opposite direction of the velocity v, we then estimate the damping coefficient c. Finally, Q of the plate can be obtained as

$$Q = \frac{2\pi m f_{\rm res}}{c}. \tag{4}$$

All the parameters used in our simulations for pyrolytic graphite plates are listed in Table I.

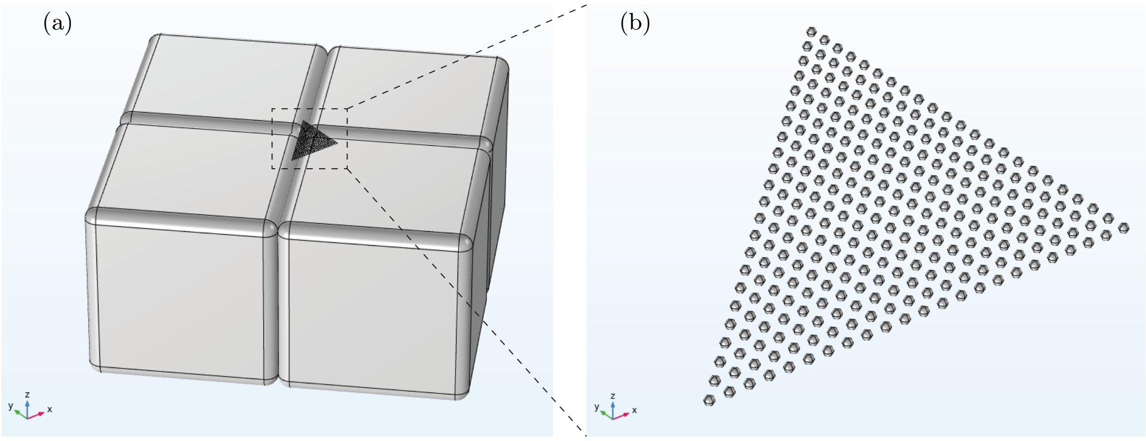

FIG. S7. COMSOL model. (a) Geometry model of magnets and graphite particles. (b) Spherical graphite particles.

S3.2 Eddy current damping in a composite plate

In this section we explain how the eddy current damping forces of a composite plate levitating above four permanent magnets are calculated. Because the magnetic susceptibility and electrical conductivity of the epoxy are negligible, the composite is modeled only by the graphite spheres to reduce the computation time, assuming that spheres are distributed homogeneously. As discussed in the main text, to account for experimental deviations from the theoretical model due to variations in particle size, composition, morphology and distribution, we use $C_\mathrm{r}d$ as the effective particle size in the simulation for particles with mean size of d, where $C_\mathrm{r} = 6.3$ is an effective particle size factor. Fig. S7a shows the geometry model of four permanent magnets and 1/8 fraction of the graphite particles. The simulation procedure is similar to that of graphite plates. The parameters used in our simulations for the composite plates are given in Table II.

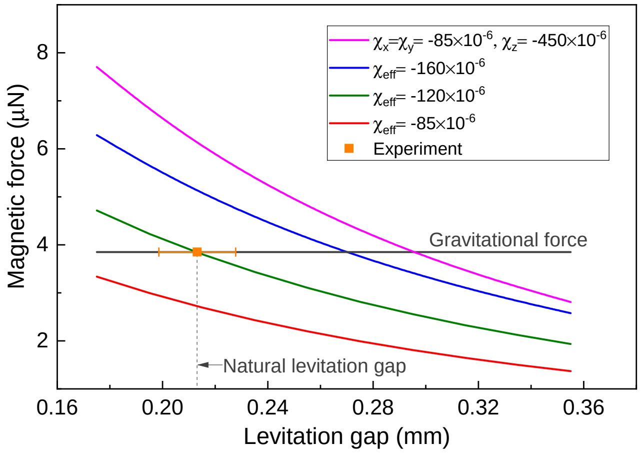

FIG. S8. Magnetic force as a function of levitation gap obtained by COMSOL simulations using different susceptibilities (solid lines), and the measured natural levitation gap (dot).

We note that graphite is inherently anisotropic [2]. However, in our fabrication procedure graphite particles are randomly oriented in the epoxy matrix, and thus by considering all possible orientations in the matrix, the local anisotropy can be averaged out and the effective macroscopic behavior can be viewed isotropic. For this reason , in our study we treat the magnetic susceptibility of graphite as an effective value χ_eff which we evaluate by fitting our FEM simulations to the measured levitation height of the composite from experiments. In Fig. S8 we showcase how χ_eff is estimated from experiments. We first measure the natural levitation gap of the composite using the Keyence microscope. By knowing the natural levitation gap and the gravitational force we then use our FEM model to estimate the magnetic force for different values of χ_eff, and find the best value that fits the experimental finding. In Fig. S8 we

show this procedure for a composite plate with $1.8 \times 1.8 \times 0.09\,\mathrm{mm}^3$, with volume fraction $V_\mathrm{f} = 0.21$ and particle size $d = 17.6\,\mu\mathrm{m}$. It can be seen that the green line which matches our experimental result is below the upper bound of the magnetic forces evaluated considering anisotropic magnetic susceptibilities, and the evaluated effective magnetic susceptibility $\chi_\mathrm{eff} = -120 \times 10^{-6}$ agrees well with the reported value in reference [4].

TABLE II. Material properties of the graphite particles and epoxy used for the simulations of the levitating composite plates.

Property	Symbol	Value	Unit
Graphite density	ρ_g	2250	$\mathrm{kg/m^3}$
Epoxy density	ρ_e	1100	$\mathrm{kg/m^3}$
Graphite susceptibility	χ_eff	-120	$\times 10^{-6}$
Graphite resistivity[3]	ρ_r	1/200000	Ω m

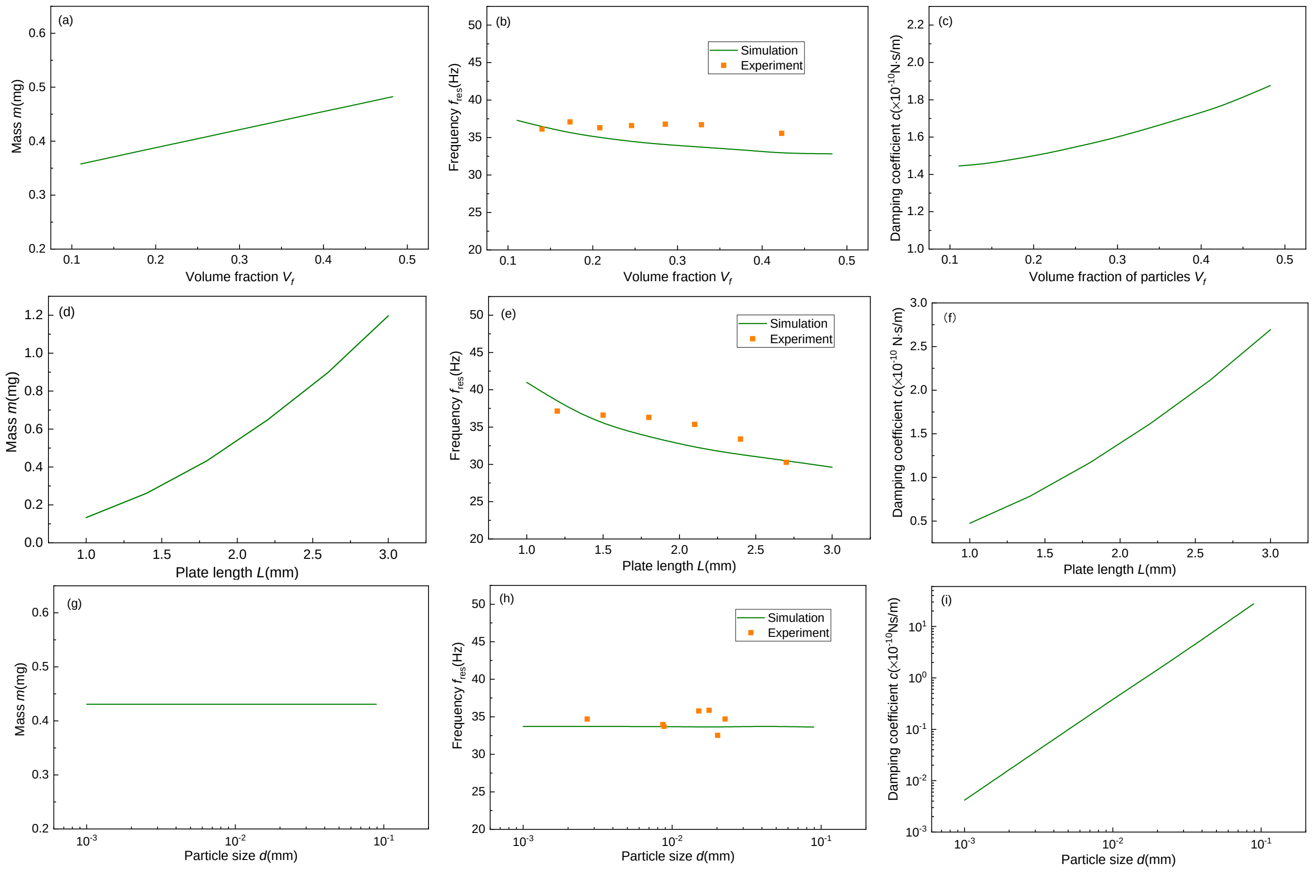

FIG. S9. (a-c) Changes in mass m, resonance frequency f_res and damping coefficient c of $1.8 \times 1.8 \times 0.09\,\mathrm{mm}^3$ composite plates with d=17.6 μm particles and different volume fraction V_f; (d-f) Changes in mass m, resonance frequency f_res and damping coefficient c of composite plates with d=17.6 μm particles and volume fraction $V_\mathrm{f} = 0.21$, but different plate length L; (g-i) Changes in mass m, resonance frequency f_res and damping coefficient c of $1.8 \times 1.8 \times 0.09\,\mathrm{mm}^3$ composite plates with volume fraction $V_\mathrm{f} = 0.32$ and different particle size d. The lines correspond to data obtained from COMSOL simulations and dots represent measured data.

Fig. S9a-c show the change of mass m, resonance frequency f_res and damping coefficient c of composite plates with different particle volume fraction V_f. Fig. S9d-f show the change of mass m, resonance frequency f_res and damping coefficient c of composite plates with different plate length L. Fig. S9g-i show the change of mass m, resonance frequency f_res and damping coefficient c of composite plates with different particle size d.

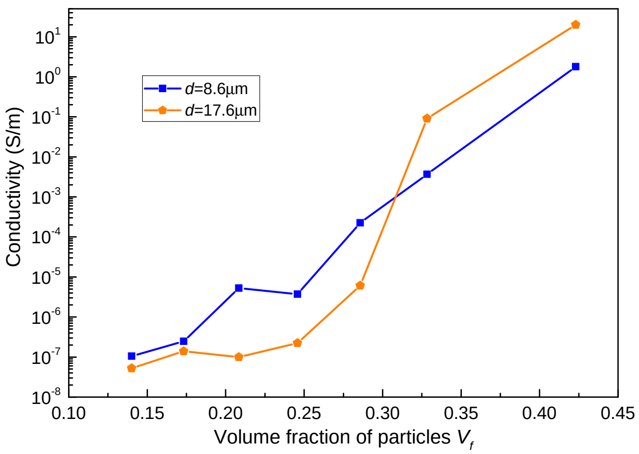

FIG. S10. Conductivity of two composites as a function of volume fraction.

S4: ELECTRICAL CONDUCTIVITY OF THE COMPOSITES

Fig. S10 shows the bulk conductivity of two composites made from $d = 8.6\,\mu\text{m}$ and $d = 17.6\,\mu\text{m}$ particles with different volume fractions. The conductivity is measured by the two-point measurement method with Agilent 4263B LCR meter (Santa Clara, CA, USA). It can be seen from the figure that the bulk conductivity of the composite is increasing with higher graphite volume fractions.

S5: ANALYTICAL MODELLING OF EDDY CURRENT DAMPING

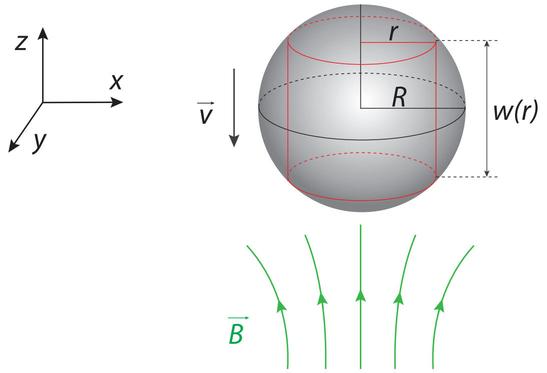

FIG. S11. A sphere model.

In this section we obtain the Q-factor of a diamagnetic particle moving in a magnetic field, analytically. We consider a spherical particle with radius $R = d/2$ that moves inside a magnetic field $\vec{B}$ as shown in Fig. S11. To calculate the eddy current loss of the sphere, we assume the sphere consists of cylinders with varying radius r and height $w(r)$ as shown in Fig. S11. It is also assumed that the sphere is small compared to the magnetic field distribution and that the magnetic field $\mathbf{B}$ is not changing in different locations on the sphere. According to Faraday's law of induction, the induced voltage on each ring ($\mathrm{d}r$) of the sphere can then be calculated as [5]:

$$emf = \frac{\mathrm{d}\Phi}{\mathrm{d}t} = \frac{\mathrm{d}(\pi r^2 B)}{\mathrm{d}t} = \pi r^2 \frac{\mathrm{d}B}{\mathrm{d}z}\frac{\mathrm{d}z}{\mathrm{d}t} = \pi r^2 v \frac{\mathrm{d}B}{\mathrm{d}z}, \tag{5}$$

where Φ is the magnetic flux, and v is the velocity of the sphere. The induced current in the ring will then be

$$\mathrm{d}I = \pi r^2 v \frac{\mathrm{d}B}{\mathrm{d}z}\frac{w(r)\mathrm{d}r}{2\pi r \rho_\mathrm{r}}, \tag{6}$$

where ρ_r is the electrical resistivity of the sphere. Using Eq. (5) and (6), the power loss in the ring can then be calculated as

$$\mathrm{d}P = \pi r^3 v^2 \left(\frac{\mathrm{d}B}{\mathrm{d}z}\right)^2 \frac{w(r)\mathrm{d}r}{2\rho_\mathrm{r}}, \tag{7}$$

from which the total loss inside the sphere will become

$$P = \pi v^2 \left(\frac{\mathrm{d}B}{\mathrm{d}z}\right)^2 \frac{1}{2\rho_\mathrm{r}} \int_0^R r^3 2\sqrt{R^2 - r^2}\mathrm{d}r = \frac{2\pi v^2 R^5}{15\rho_\mathrm{r}} \left(\frac{\mathrm{d}B}{\mathrm{d}z}\right)^2. \tag{8}$$

Finally, the eddy current loss per unit volume of the sphere becomes

$$P_\mathrm{unit} = \frac{v^2 R^2}{10\rho_\mathrm{r}} \left(\frac{\mathrm{d}B}{\mathrm{d}z}\right)^2. \tag{9}$$

Considering $P_\mathrm{unit} = F_\mathrm{eddy} v = c_\mathrm{unit} v^2$, and using Eq. 9, the damping coefficient c_unit can then be expressed as

$$c_\mathrm{unit} = \frac{R^2}{10\rho_\mathrm{r}} \left(\frac{\mathrm{d}B}{\mathrm{d}z}\right)^2. \tag{10}$$

Which results in the following expression for the Q-factor solely due to eddy currents:

$$Q_\mathrm{sphere} = \frac{2\pi m_\mathrm{sphere} f_\mathrm{res}}{c_\mathrm{sphere}} = \frac{2\pi \rho_\mathrm{d} V_\mathrm{sphere} f_\mathrm{res}}{c_\mathrm{unit} V_\mathrm{sphere}} = \frac{2\pi \rho_\mathrm{d} f_\mathrm{res}}{c_\mathrm{unit}} = \frac{20\pi f_\mathrm{res} \rho_\mathrm{d} \rho_\mathrm{r}}{R^2 (\mathrm{d}B/\mathrm{d}z)^2}, \tag{11}$$

in which m_sphere is the mass of the particle, f_res is the resonance frequency, ρ_d is the density and V_sphere is the volume of the sphere.

For a composite plate consisting of spheres of radius R dispersed in an insulating matrix, the Q can be determined similar to Eq.(11) as follows:

$$Q_\mathrm{plate} = \frac{2\pi m_\mathrm{plate} f_\mathrm{res}}{c_\mathrm{plate}} = \frac{2\pi \rho_\mathrm{p} V_\mathrm{plate} f_\mathrm{res}}{\int_{V_\mathrm{spheres}} c_\mathrm{unit} \mathrm{d}V_\mathrm{spheres}} = \frac{20\pi f_\mathrm{res} \rho_\mathrm{p} \rho_\mathrm{r} V_\mathrm{plate}}{R^2 \int_{V_\mathrm{spheres}} \left(\frac{\mathrm{d}B}{\mathrm{d}z}\right)^2 \mathrm{d}V_\mathrm{spheres}}, \tag{12}$$

where ρ_p is the density of the composite plate, V_plate is the volume of the plate, and V_spheres is the volume of the spherical particles. For a graphite/epoxy composite plate with volume fraction of V_f, the Q is then

$$Q_\mathrm{plate} = \frac{20\pi f_\mathrm{res} \rho_\mathrm{r} V_\mathrm{plate} (V_\mathrm{f}(\rho_\mathrm{g} - \rho_\mathrm{e}) + \rho_\mathrm{e})}{(C_\mathrm{r} R)^2 \int_{V_\mathrm{spheres}} \left(\frac{\mathrm{d}B}{\mathrm{d}z}\right)^2 \mathrm{d}V_\mathrm{spheres}} = \frac{20\pi f_\mathrm{res} \rho_\mathrm{r} V_\mathrm{plate} (V_\mathrm{f}(\rho_\mathrm{g} - \rho_\mathrm{e}) + \rho_\mathrm{e})}{(C_\mathrm{r} R)^2 \nabla^2 B V_\mathrm{f} V_\mathrm{plate}} = \frac{20\pi f_\mathrm{res} \rho_\mathrm{r} ((\rho_\mathrm{g} - \rho_\mathrm{e}) + \rho_\mathrm{e}/V_\mathrm{f})}{(C_\mathrm{r} R)^2 \nabla^2 B}, \tag{13}$$

where ρ_g is the density of graphite, ρ_e is the density of epoxy, and C_r is the apparent particle size factor that accounts for the uncertainties related to particle size, morphology and distribution. And

$$\nabla^2 B = \frac{\int_{V_\mathrm{plate}} \left(\frac{\mathrm{d}B}{\mathrm{d}z}\right)^2 \mathrm{d}V_\mathrm{plate}}{V_\mathrm{plate}}, \tag{14}$$

that can be obtained numerically using Comsol.

S6: Q-FACTORS AND ACCELERATION NOISE FLOOR OF STATE-OF-THE-ART LEVITODYNAMIC SYSTEMS

In Table III - V we list the Qs and acceleration noise floors $\sqrt{S_\mathrm{aa}}$ of different levitodynamic systems showed in Fig. 5 of the main text. In Table III, the Qs were measured directly without feedback cooling either at room temperature or cryogenic temperature. In Table IV, we show the Qs measured at room temperature with feedback cooling and the natural Qs estimated at room temperature without feedback cooling. In Table V, the reported acceleration noise floor of different levitodynamic systems under different measurement conditions are listed. The data marked with [∗] are estimated using the following equation [6]:

$$\sqrt{S_{aa}} = \sqrt{\frac{8\pi f_\mathrm{res} k_\mathrm{B} T}{mQ}} \tag{15}$$

where f_res is the resonance frequency, m is the mass, Q is the quality factor, T is the temperature and k_B is the Boltzmann constant.

TABLE III. Q-factors of different levitodynamic systems without feedback cooling (RT: room temperature).

mass(kg)	Q	Levitation method	Temperature	Reference
3.3×10^{-18}	1.0×10^{7}	optical	RT	[7]
3.7×10^{-14}	2.1×10^{4}		RT	[8]
9.6×10^{-17}	1.5×10^{6}	electrical	RT	[9]
6.1×10^{-10}	1.3×10^{7}	superconducting	4.2K	[10]
1.1×10^{-10}	1.0×10^{6}		<90K	[11]
5.7×10^{-11}	5.0×10^{4}		5K	[12]
4.0×10^{-6}	5.5×10^{3}		5K	[6]
2.7×10^{-13}	2.0×10^{7}	diamagnetic	3K	[13]
7.8×10^{-8}	1.5×10^{5}		RT	[14]
1.0×10^{-5}	362		RT	[15]
2.3×10^{-5}	176		RT	[15]
3.9×10^{-5}	115		RT	[15]
6.3×10^{-5}	76		RT	[15]
2.3×10^{-6}	4.6×10^{5}		RT	this work

TABLE IV. Q-factors of different levitodynamic systems with feedback cooling.

mass(kg)	With feedback cooling		Estimated natural damping		Levitation method	Ref
	Q	Effective temperature	Q	Effective temperature		
1.4×10^{-19}	440	3mK	4.4×10^{7}	300K	optical	[16]
3.1×10^{-17}	38.6	460mK	2.5×10^{4}	300K	optical	[17]
3.0×10^{-14}	14.8	10K	445	300K	optical	[18]
2.5×10^{-10}	175	9K	5.8×10^{3}	300K	diamagnetic	[19]
3.1×10^{-15}	13.7	1.2mK	3.2×10^{6}	295K		[20]

TABLE V. Acceleration noise floor of different levitodynamic systems showed in Fig. 5 of the main text. Data marked with [∗] represent the estimated value.

mass(kg)	$\sqrt{S_{\mathrm{aa}}}(\mathrm{g}/\sqrt{\mathrm{Hz}})$	levitation method	Effective temperature	Reference	Note
3.3×10^{-18}	1.1×10^{-3}	optical	RT	[7]	∗
3.7×10^{-14}	1.2×10^{-4}		RT	[8]	∗
1.4×10^{-19}	3.9×10^{-3}		3mK	[16]	feedback cooling, ∗
3.1×10^{-17}	5.4×10^{-3}		460mK	[17]	feedback cooling
3.0×10^{-14}	7.0×10^{-4}		10K	[18]	feedback cooling
1.4×10^{-18}	2.3×10^{-3}		3K	[21]	feedback cooling
8.4×10^{-14}	1.2×10^{-5}		∼	[22]	feedback cooling
1.4×10^{-13}	3.6×10^{-5}		1K	[23]	feedback cooling
1.5×10^{-13}	7.5×10^{-6}		∼mK	[24]	feedback cooling
1.2×10^{-11}	4.0×10^{-7}		∼	[25]	feedback cooling
9.4×10^{-13}	9.5×10^{-8}		50 μK	[26]	feedback cooling
9.6×10^{-17}	2.4×10^{-5}	electrical	RT	[9]	∗
6.1×10^{-10}	8.5×10^{-10}	superconducting	4.2K	[10]	∗
1.1×10^{-10}	5.0×10^{-8}		<90K	[11]	∗
5.7×10^{-11}	1.6×10^{-8}		5K	[12]	∗
4.0×10^{-6}	1.2×10^{-10}		5K	[6]	∗
2.7×10^{-13}	4.8×10^{-9}	diamagnetic	3K	[13]	∗
7.8×10^{-8}	9.7×10^{-10}		RT	[14]	-
2.5×10^{-10}	3.6×10^{-8}		9K	[19]	feedback cooling
3.1×10^{-15}	3.1×10^{-6}		1.2mK	[20]	feedback cooling, ∗
1.0×10^{-5}	2.6×10^{-9}		RT	[15]	∗
2.3×10^{-5}	2.3×10^{-9}		RT	[15]	∗
3.9×10^{-5}	2.1×10^{-9}		RT	[15]	∗
6.3×10^{-5}	1.9×10^{-9}		RT	[15]	∗
2.3×10^{-6}	1.6×10^{-10}		RT	this work	∗

[1] D. Bray, S. Gilmour, F. Guild, T. Hsieh, K. Masania, and A. Taylor, Quantifying nanoparticle dispersion: application of the delaunay network for objective analysis of sample micrographs, Journal of materials science **46**, 6437 (2011).

[2] M. Simon and A. Geim, Diamagnetic levitation: Flying frogs and floating magnets, Journal of applied physics **87**, 6200 (2000).

[3] J. Pappis and S. Blum, Properties of pyrolytic graphite, Journal of the American Ceramic Society **44**, 592 (1961).

[4] M. Boukallel, J. Abadie, and E. Piat, Levitated micro-nano force sensor using diamagnetic materials, in *2003 IEEE International Conference on Robotics and Automation (Cat. No. 03CH37422)*, Vol. 3 (IEEE, 2003) pp. 3219–3224.

[5] A. Taghvaei, H. Shokrollahi, K. Janghorban, and H. Abiri, Eddy current and total power loss separation in the iron–phosphate–polyepoxy soft magnetic composites, Materials & Design **30**, 3989 (2009).

[6] C. Timberlake, G. Gasbarri, A. Vinante, A. Setter, and H. Ulbricht, Acceleration sensing with magnetically levitated oscillators above a superconductor, Applied Physics Letters **115**, 224101 (2019).

[7] J. Gieseler, B. Deutsch, R. Quidant, and L. Novotny, Subkelvin parametric feedback cooling of a laser-trapped nanoparticle, Physical review letters **109**, 103603 (2012).

[8] T. Li, S. Kheifets, and M. G. Raizen, Millikelvin cooling of an optically trapped microsphere in vacuum, Nature Physics **7**, 527 (2011).

[9] A. Pontin, N. Bullier, M. Toroš, and P. Barker, Ultranarrow-linewidth levitated nano-oscillator for testing dissipative wave-function collapse, Physical Review Research **2**, 023349 (2020).

[10] A. Vinante, P. Falferi, G. Gasbarri, A. Setter, C. Timberlake, and H. Ulbricht, Ultralow mechanical damping with meissner-levitated ferromagnetic microparticles, Physical Review Applied **13**, 064027 (2020).

[11] J. Gieseler, A. Kabcenell, E. Rosenfeld, J. Schaefer, A. Safira, M. J. Schuetz, C. Gonzalez-Ballestero, C. C. Rusconi, O. Romero-Isart, and M. D. Lukin, Single-spin magnetomechanics with levitated micromagnets, Physical Review Letters **124**, 163604 (2020).

[12] T. Wang, S. Lourette, S. R. O'Kelley, M. Kayci, Y. Band, D. F. J. Kimball, A. O. Sushkov, and D. Budker, Dynamics of a ferromagnetic particle levitated over a superconductor, Physical Review Applied **11**, 044041 (2019).

[13] Y. Leng, R. Li, X. Kong, H. Xie, D. Zheng, P. Yin, F. Xiong, T. Wu, C.-K. Duan, Y. Du, *et al.*, Mechanical dissipation below 1 μ hz with a cryogenic diamagnetic levitated micro-oscillator, Physical Review Applied **15**, 024061 (2021).

[14] F. Xiong, P. Yin, T. Wu, H. Xie, R. Li, Y. Leng, Y. Li, C. Duan, X. Kong, P. Huang, and J. Du, Lens-free optical detection of thermal motion of a submillimeter sphere diamagnetically levitated in high vacuum, Phys. Rev. Applied **16**, L011003 (2021).

[15] X. Chen, A. Keşkekler, F. Alijani, and P. G. Steeneken, Rigid body dynamics of diamagnetically levitating graphite resonators, Applied Physics Letters **116**, 243505 (2020).

[16] J. Vovrosh, M. Rashid, D. Hempston, J. Bateman, M. Paternostro, and H. Ulbricht, Parametric feedback cooling of levitated optomechanics in a parabolic mirror trap, JOSA B **34**, 1421 (2017).

[17] G. Ranjit, M. Cunningham, K. Casey, and A. A. Geraci, Zeptonewton force sensing with nanospheres in an optical lattice, Physical Review A **93**, 053801 (2016).

[18] G. Ranjit, D. P. Atherton, J. H. Stutz, M. Cunningham, and A. A. Geraci, Attonewton force detection using microspheres in a dual-beam optical trap in high vacuum, Physical Review A **91**, 051805 (2015).

[19] C. W. Lewandowski, T. D. Knowles, Z. B. Etienne, and B. D'Urso, High-sensitivity accelerometry with a feedback-cooled magnetically levitated microsphere, Physical Review Applied **15**, 014050 (2021).

[20] B. R. Slezak, C. W. Lewandowski, J.-F. Hsu, and B. D'Urso, Cooling the motion of a silica microsphere in a magneto-gravitational trap in ultra-high vacuum, New Journal of Physics **20**, 063028 (2018).

[21] D. Hempston, J. Vovrosh, M. Toroš, G. Winstone, M. Rashid, and H. Ulbricht, Force sensing with an optically levitated charged nanoparticle, Applied Physics Letters **111**, 133111 (2017).

[22] A. Kawasaki, A. Fieguth, N. Priel, C. P. Blakemore, D. Martin, and G. Gratta, High sensitivity, levitated microsphere apparatus for short-distance force measurements, Review of Scientific Instruments **91**, 083201 (2020).

[23] D. C. Moore, A. D. Rider, and G. Gratta, Search for millicharged particles using optically levitated microspheres, Physical review letters **113**, 251801 (2014).

[24] A. D. Rider, C. P. Blakemore, G. Gratta, and D. C. Moore, Single-beam dielectric-microsphere trapping with optical heterodyne detection, Physical Review A **97**, 013842 (2018).

[25] F. Monteiro, S. Ghosh, A. G. Fine, and D. C. Moore, Optical levitation of 10-ng spheres with nano-g acceleration sensitivity, Physical Review A **96**, 063841 (2017).

[26] F. Monteiro, W. Li, G. Afek, C.-l. Li, M. Mossman, and D. C. Moore, Force and acceleration sensing with optically levitated nanogram masses at microkelvin temperatures, Physical Review A **101**, 053835 (2020).
